# Retraction Note: Experimental design and analysis of advanced three phase converter for PV application with WCO-P&O MPPT controller

**DOI:** 10.1038/s41598-025-93000-w

**Published:** 2025-03-11

**Authors:** K. Krishnaram, S. Sivamani, Zuhair Alaas, M. M. R. Ahmed, S. Senthilkumar, S. Antony Raj

**Affiliations:** 1 Department of Electrical and Electronics Engineering, E.G.S. Pillay Engineering College, Nagapattinam, Tamil Nadu 611002 India; 2 Department of Electrical Engineering, Faculty of Engineering, Jazan University, 45142 Jizan, Saudi Arabia; 3 Faculty of Technology and Education, Helwan University, Helwan, Egypt; 4 Department of Electrical and Electronics Engineering, St. Anns College of Engineering and Technology, Chirala, Andhra Pradesh 523187 India; 5 Department of Electronics and Communication Engineering, E.G.S. Pillay Engineering College, Nagapattinam, Tamil Nadu 611002 India

Retraction of: *Scientific Reports*, 10.1038/s41598-024-61856-z, published on 14 May 2024

The Editors have retracted this Article.

After publication, concerns were raised about the authorship of this paper. During subsequent investigation, the Authors were unable to provide the original code and data the Article is based on. Additionally, Fig. 19 appears to be very similar to Fig. 9 in a previously-published article by different authors [1]. The Authors stated that they used the same laboratory; the laboratory, however, did not confirm this. The Editors no longer have confidence in the veracity of the data presented in this Article.

Zuhair Alaas and M. M. R. Ahmed agree with this retraction and its wording. K. Krishnaram, S. Sivamani, S. Senthilkumar and S. Antony Raj disagree with the retraction.
